# Dose reduction and discontinuation of sacubitril/valsartan over time in patients undergoing maintenance hemodialysis

**DOI:** 10.3389/fcvm.2026.1804910

**Published:** 2026-08-12

**Authors:** Ying Guo, Min Yin, Qianyu Li, Shuojie Guo, Li Zhang

**Affiliations:** 1 Department of Nephrology, China-Japan Union Hospital of Jilin University, Changchun, China; 2 Department of Neurology, China-Japan Union Hospital of Jilin University, Changchun, China

**Keywords:** antihypertensives, end-stage renal disease, hemodialysis, intradialytic hypotension, sacubitril/valsartan

## Abstract

**Objective:**

This study aims to evaluate the therapeutic effects and dose adjustment patterns of sacubitril/valsartan (SV) over time in patients undergoing maintenance hemodialysis (HD).

**Methods:**

The clinical data of end-stage renal disease patients receiving maintenance HD at our center were retrospectively reviewed. A total of 151 patients treated with SV were included. All patients were followed up regularly in the dialysis outpatient clinic. The clinical characteristics, biochemical parameters, and echocardiographic measurements were collected at baseline and during follow-up. The efficacy, safety, and dose adjustment patterns of SV therapy were analyzed.

**Results:**

A six-month observation period was selected for analysis to ensure adequate follow-up while maximizing the number of eligible patients included in the study. SV dose reduction was observed in 62 patients, and 48 patients had discontinued concomitant antihypertensive medications. The greatest reduction in the proportion of patients receiving SV combined with other antihypertensive agents occurred at the fourth month after dialysis initiation (39.8%). Patients receiving SV without dose reduction and in combination with other antihypertensive drugs had higher interdialytic and intradialytic blood pressure, when compared with patients receiving SV monotherapy or reduced/discontinued SV. The multivariate analysis identified diabetes mellitus [odds ratio [OR]: 3.085, 95% confidence interval [CI]: 1.137–8.376, *p* = 0.027], higher baseline N-terminal pro B-type natriuretic peptide (OR: 1.870, 95% CI: 1.109–3.152, *p* = 0.019), and higher interdialytic weight gain-to-dry weight ratio (OR: 4.083, 95% CI: 1.763–9.457, *p* = 0.001) as independent predictors of intradialytic hypotension. During follow-up, six composite events occurred in the SV combination or monotherapy group, while 13 composite events occurred in the SV dose reduction or discontinuation group. There was no significant difference between groups.

**Conclusion:**

In real-world practice, the dose reduction or discontinuation of SV is common in HD patients. Clinicians should carefully balance individual risks and benefits, apply SV with caution, and closely monitor patients receiving this therapy.

## Introduction

Chronic kidney disease is a progressive condition characterized by the gradual decline in kidney function, ultimately leading many patients to end-stage renal disease (ESRD) that requires renal replacement therapy (1). ESRD patients undergoing maintenance hemodialysis (HD) face the substantial burden of cardiovascular disorders (2). Among these complications, heart failure (HF) is the leading cause of mortality and rehospitalization (3). The prevalence of HF in the HD population is estimated to be 30%–50%, and the 5-year survival rate remains below 40% (4). Several factors contribute to this elevated risk, including long-standing hypertension, recurrent volume overload, myocardial fibrosis, and chronic inflammation (5). In addition, the rapid hemodynamic and metabolic fluctuations inherent to HD can further compromise cardiac performance, making HF management in this population particularly challenging (6).

Sacubitril/valsartan (SV), which is the first angiotensin receptor-neprilysin inhibitor, has transformed HF treatment in the general population. By inhibiting neprilysin, SV enhances the natriuretic peptide system, promoting diuresis, vasodilation, and antifibrotic effects, while simultaneously blocking the angiotensin II type 1 receptor to suppress the overactivation of the renin-angiotensin-aldosterone system. This dual mechanism provides substantial clinical benefits. As reported in the PARADIGM-HF trial, SV significantly improved the survival in non-dialysis HF patients (7). Furthermore, SV has demonstrated superior cardioprotective effects in HF patients with reduced ejection fraction, significantly lowering both cardiovascular and all-cause mortality (8–10). However, it remains to be explored whether SV can offer similar benefits for patients undergoing HD.

Several recent studies have reported the favorable effects of SV on blood pressure (BP) control, cardiac structure and function, and clinical outcomes in HD patients with hypertension and HF (11, 12). However, since HD patients were excluded from most pivotal clinical trials, high-quality evidence that supports SV use in this population remains limited (13, 14). The present study aimed to explore the implications of SV dose adjustment in maintenance HD patients. A cohort of HD patients receiving SV was examined to characterize the longitudinal treatment effects, identify the clinical factors associated with dose reduction or discontinuation, and evaluate the potential impact of these adjustments on BP, cardiac structure and function, and clinical outcomes. These findings may provide direct real-world evidence to inform individualized SV dosing strategies in HD patients.

## Methods

### Study design and participants

The present single-center retrospective study was conducted at the Hemodialysis Center of China-Japan Union Hospital, Jilin University. Patients who began dialysis induction at our center between September 2019 and September 2024 were eligible for enrollment. The inclusion criteria were, as follows: (1) patients undergoing maintenance HD, 2–3 times per week, with each session lasting 3.5–4.0 h; (2) a diagnosis of hypertension prior to initiation of HD; (3) initiation of SV therapy before dialysis, with continued oral SV for more than one month after starting dialysis at doses of 100–400 mg/day, either alone or in combination with other antihypertensive medications; (4) follow-up duration exceeding six months. The third criterion was designed to ensure a relatively stable SV exposure at the time of dialysis initiation, and minimize confounding related to acute medication adjustments during the early dialysis transition period. At the center of the investigators, many patients with advanced CKD were already prescribed SV for heart failure or refractory hypertension before dialysis initiation, reflecting a common real-world clinical scenario.

The exclusion criteria were, as follows: (1) patients with acute coronary syndrome or renal artery stenosis; (2) patients with irregular dialysis; (3) patients with severe valvular heart disease or arrhythmias; (4) patients who are unable to attend follow-up visits. A total of 350 patients who initiated HD were screened. Among these patients, 151 patients met the enrollment criteria, which included 86 male and 65 female patients.

In order to minimize selection and information bias, strict inclusion and exclusion criteria were applied, and all data were extracted using a standardized protocol. Clinical data were independently cross-checked by two investigators to ensure accuracy. BP measurements were performed by a fixed dialysis care team using standardized procedures to reduce inter-observer variability. Echocardiographic examinations were conducted by experienced operators at the center of the investigators, and all laboratory measurements were performed at the institutional laboratory of the investigators using standardized assays, thereby minimizing measurement bias.

The study protocol was approved by the Ethics Committee of China-Japan Union Hospital, Jilin University (Approval no. 2026052502).

### Data collection

The data were retrospectively collected from medical records. The demographic characteristics, comorbidities at dialysis initiation [*i.e.,* atherosclerotic cardiovascular disease, cancer, chronic lung disease, cerebrovascular disease, diabetes mellitus (DM), and peripheral vascular disease], smoking status, and alcohol consumption were recorded. The clinical data and laboratory results included the New York Heart Association (NYHA) functional class, estimated glomerular filtration rate (eGFR), parathyroid hormone (PTH), N-terminal pro B-type natriuretic peptide (NT-proBNP), serum albumin, potassium, calcium, and phosphorus. The eGFR was calculated using the formula proposed by the Chronic Kidney Disease Epidemiology Collaboration 2021 (15).

Two-dimensional echocardiography was performed by experienced sonographers within two weeks before and after the initiation of SV therapy, and during follow-up. The echocardiographic measurements were obtained with patients in the left lateral decubitus position, synchronized with electrocardiography, averaging three consecutive cardiac cycles for sinus rhythm or five cycles for arrhythmias. The left atrial anteroposterior diameter (LAd), left ventricular end-diastolic diameter (LVEDd), and interventricular septal thickness (IVSd) were measured in the parasternal long-axis view. The right atrial diameter (RAd) and right ventricular end-diastolic diameter (RVDd) were measured in the apical four-chamber view. The left ventricular ejection fraction (LVEF) was determined using the Simpson method.

All included patients completed the six-month observation period, with no loss to follow-up. Key variables, including BP, NT-proBNP, and echocardiographic parameters, were fully available (100%). For laboratory variables with occasional missing values, multiple imputation was applied where appropriate, while categorical variables with missing rates of <5% were analyzed using complete-case methods. Medication adherence was assessed through regular (1–2 times weekly) patient interviews, combined with longitudinal BP trends during dialysis sessions, and patients with poor adherence (<80%) were excluded from the analysis.

### Follow-up and outcome measures

During the six-month observation period, the following events were recorded. Hypotension was defined and classified into intradialytic, interdialytic, and orthostatic subtypes. Intradialytic hypotension (IDH) refers to the decrease in systolic blood pressure (SBP) of ≥20 mmHg or reduction in mean arterial pressure of ≥10 mmHg during dialysis, accompanied by hypotensive symptoms (16). Interdialytic hypotension was defined through daily BP monitoring on non-dialysis days, with SBP consistently below 100 mmHg (17). Orthostatic hypotension was defined as a fall in SBP of ≥20 mmHg and/or a diastolic blood pressure (DBP) of ≥10 mmHg within three minutes of standing, or during a tilt-table test with ≥60° inclination, with or without hypoperfusion symptoms. For patients with supine hypertension (SBP ≥150 mmHg or DBP ≥90 mmHg), a decrease in SBP of ≥30 mmHg upon standing within three minutes was considered diagnostic (18). “Baseline SBP” was defined as the pre-dialysis SBP measured on the day of the first HD session, while “SBP prior to SV initiation” referred to the outpatient SBP recorded before the first administration of SV, typically several weeks or months before dialysis initiation.

BP was measured following the standard procedure. During dialysis, five measurements were obtained at predefined timepoints (at initiation, one hour, two hours, and three hours, and immediately before termination), and the mean values were calculated. On interdialytic days, patients performed home BP monitoring using their own upper-arm automated BP monitors certified according to ESH/ISO/BHS standards. Every six months, patients brought their devices to the dialysis center, where the readings were compared with those obtained using a standard mercury sphygmomanometer to verify measurement accuracy. Measurements were obtained after at least five minutes of rest in a seated position, with patients remaining in the supine position throughout each measurement to ensure consistency with the intradialytic measurements. On dialysis days, BP was measured in the morning and evening. On non-dialysis days, measurements were obtained in the morning, midday, and evening. Each measurement was taken twice and averaged. All patients completed standardized home BP log sheets and submitted these at each dialysis visit. If patients retained the original records, copies were documented by the research team. Adherence to home BP monitoring was assessed throughout the 6-month follow-up period, and patients who completed fewer than 80% of the required home BP measurements were considered non-adherent, and excluded from the analysis.

HF were diagnosed based on the following: (1) NYHA functional class II-IV; (2) the criteria defined in the 2021 European Society of Cardiology (ESC) guidelines, including heart failure with reduced ejection fraction (HFrEF), preserved ejection fraction (HFpEF), and mildly reduced ejection fraction (HFmrEF); (3) elevated natriuretic peptide levels, defined as NT-proBNP ≥125 ng/L or BNP ≥35 ng/L.

### IDH management

The acute management of IDH included the suspension of ultrafiltration, administration of normal saline, or intravenous injection of hypertonic glucose. Long-term strategies consisted of individualized therapeutic adjustments, including dry weight reassessment, antihypertensive regime modification, optimization of cardiac function, use of cool dialysate, and regulation of ultrafiltration rate and volume, as well as patient-centered education focused on sodium restriction, interdialytic weight control, and nutritional improvement.

### Statistical analysis

Statistical analyses were performed using SPSS version 25.0 (IBM Corp., Chicago, IL, USA). The normality of continuous variables was assessed using the Shapiro–Wilk test. Data with a normal distribution were presented in mean ± standard deviation, and the differences were analyzed using *t*-test or Wilcoxon test, as appropriate. Non-normally distributed data were expressed in median (25th percentile and 75th percentile), and comparisons were performed using Wilcoxon signed-rank test. Categorical variables were compared using *χ*^2^ test or Fisher's exact test. Longitudinal changes in interdialytic and intradialytic BP were analyzed using generalized estimating equations, and estimated marginal means (EMMs) were derived from these models to present the model-adjusted group averages over time. The EMMs accounted for the effects of time, treatment strategy, and its interaction, allowing for fair comparisons between groups, while controlling for confounding variables. Univariate and multivariate logistic regression models were used to identify the independent risk factors for IDH. The odds ratios (ORs) and 95% confidence intervals (CIs) were reported. Variables included in the multivariable logistic regression were selected based on the univariate analysis (*p* < 0.1). A two-sided *p*-value of <0.05 was considered statistically significant. A formal sensitivity analysis was not performed due to the retrospective design of the study and sample size limitations. No adjustment for multiple comparisons was applied. However, longitudinal analyses were performed using generalized estimating equations, which accounted for within-subject correlations across repeated measures.

## Results

### Study cohort

A total of 151 HD patients receiving SV between September 2019 and September 2024 were included. All patients were newly initiated on HD, and started SV treatment before dialysis initiation. The median interval between SV initiation and HD initiation was 0.5 months (IQR: 0.2–1.0 months), indicating that most patients began SV treatment shortly before starting dialysis.

The baseline characteristics of these patients are summarized in Table 1. The mean age of the cohort was 58.08 ± 9.89 years old, and 56.95% were male. In addition to hypertension, DM was the most common comorbidity, which was present in 49.67% of cases. Most patients (94.04%) began dialysis during hospitalization. At the time of dialysis initiation, the majority of patients (87.42%) were classified as NYHA functional class II-IV, with a mean eGFR of 6.11 ± 1.15 mL/min/1.73 m^2^.

**Table 1:** Baseline characteristics of the study cohort.

Variables	Total (*n* = 151)
Age	58.08 ± 9.89
Sex (male%)	86 (56.95%)
Smoking history	45 (29.80%)
Alcohol consumption history	27 (17.88%)
Comorbidities	
DM	75 (49.67%)
Chronic lung disease	3 (1.99%)
Coronary artery disease	56 (37.08%)
Cerebrovascular disease	29 (19.21%)
Peripheral vascular disease	5 (3.31%)
Malignancy	2 (1.32%)
eGFR (mL/min/1.73 m^2^)	6.11 ± 1.15
NYHA functional class	
Ⅰ	19 (12.58%)
Ⅱ	43 (28.48%)
Ⅲ	68 (45.03%)
Ⅳ	21 (13.91%)
Phosphorus (mmol/L)	1.89 ± 0.84
Calcium (mmol/L)	2.01 ± 0.21
Potassium (mmol/L)	4.54 ± 0.96
Sodium (mmol/L)	138.54 ± 2.80
Triglycerides	1.54 ± 0.92
Total cholesterol	3.46 ± 1.25
Low-density lipoprotein cholesterol (mmol/L)	2.13 ± 0.67
High-density lipoprotein cholesterol (mmol/L)	1.19 ± 0.45
Hemoglobin (g/L)	102.3 ± 14.27
Albumin (g/L)	35.87 ± 3.51
Parathyroid hormone (pg/mL)	174.54 (87.57−325.66)
NT-proBNP (pg/mL)	11,680 (7,640, 20,000)

DM, diabetes mellitus; eGFR, estimated glomerular filtration rate; NYHA, New York Heart Association; NT-proBNP, N-terminal pro B-type natriuretic peptide.

The initial dose of SV ranged from 100 to 400 mg/day, with an average dose of 174.8 ± 63.2 mg/day. Concomitant antihypertensive therapy was common. A total of 147 (97.4%) patients used additional medications, including calcium channel blockers (CCBs) and *β*-blockers. Seven patients received terazosin.

The median follow-up duration was 39 months (range: 8–60 months). Since the follow-up duration varied according to the timing of dialysis initiation, a predefined six-month observation period was used for the longitudinal analyses to ensure adequate follow-up while maximizing the number of eligible patients included in the study. Accordingly, all efficacy and outcome analyses were limited to this 6-month observation period.

During the observation period, medication adjustments were frequent: 62 patients underwent SV dose reduction and discontinuation, or tapering of CCBs and *β*-blockers; 48 patients discontinued all antihypertensive medications; 42 patients maintained the initial SV dose without dose reduction or discontinuation, either alone or in combination with CCBs or *β*-blockers. Notably, nine patients (15.79%) who previously discontinued the antihypertensive therapy required the re-initiation of SV due to recurrent hypertension. Among these patients, four patients (7.0%) experienced a more marked BP elevation (18.66% above baseline), and required the addition of CCB therapy alongside SV.

### BP at baseline and after SV treatment

The mean SBP before dialysis was 152.6 ± 15.0 mmHg, and the mean SBP prior to SV treatment was 152.4 ± 14.7 mmHg. No significant difference was observed between these two measurements. Since the initiation of dialysis generally coincided with the start of SV therapy, no significant difference was observed between these two baseline measurements. After six months of treatment, the mean interdialytic SBP decreased to 142.8 ± 6.0 mmHg, and the mean intradialytic SBP declined to 138.5 ± 6.7 mmHg. Compared with both the predialysis and pre-SV baseline values, SBP was significantly lower during the six-month observation period in both interdialytic and intradialytic measurements (*p* < 0.001).

### Changes in SV use over time with dialysis duration

**Figure 1:**
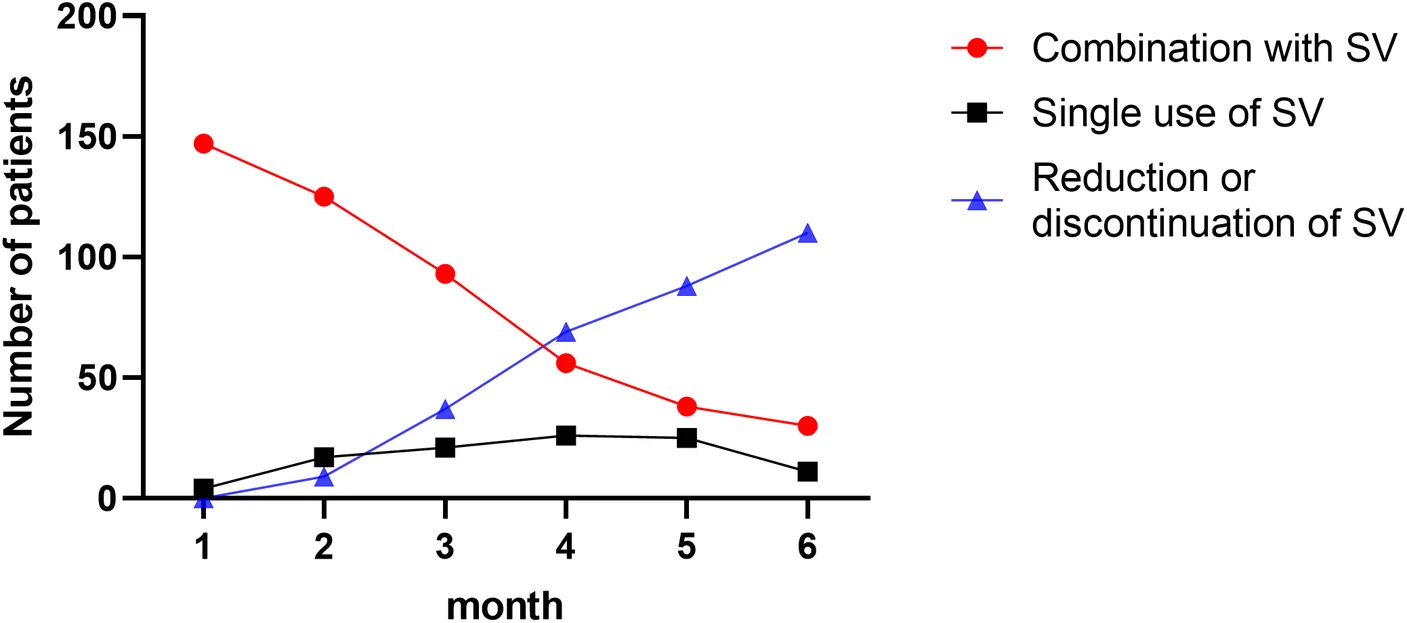
Changes in the number of patients using different antihypertensive regimens during dialysis follow-up.

The review of antihypertensive medications among the 151 patients revealed that with the increase in dialysis duration, the number of patients receiving SV in combination with other antihypertensive agents gradually declined, while the number of patients treated with SV monotherapy or undergoing SV dose reduction/discontinuation increased. Notably, after six months of follow-up, the proportion of patients whose SV dosage was reduced or discontinued (with cessation of other antihypertensive medications) markedly increased. The largest decrease in the proportion of patients receiving combination therapy occurred at month four, with a reduction of 39.8% (Figure 1).

Interdialytic SBP progressively decreased over the six-month observation period, and the mixed-effects modeling revealed the significant effects of time (Wald *χ*^2^ = 33.444, *p* < 0.001), treatment strategy (Wald *χ*^2^ = 34.226, *p* < 0.001), and its interaction (Wald *χ*^2^ = 34.002, *p* < 0.001). Compared with month one, the EMMs were significantly lower from months two through six. Patients receiving SV dose reduction or discontinuation demonstrated greater reductions in interdialytic pressure, when compared to patients receiving combination therapy (*p* < 0.001), while the differences between combination therapy and SV monotherapy did not reach statistical significance (Table 2).

**Table 2:** EMMs of interdialytic SBP (mmHg) over six months.

Follow-up months	Combination therapy	SV monotherapy	SV dose reduction/discontinuation
1	150.99 ± 1.07	144.25 ± 4.07	–
2	148.66 ± 1.11	138.65 ± 1.80^a^	134.67 ± 1.98^a^
3	146.26 ± 1.24	137.48 ± 1.30^a^	141.00 ± 1.31
4	145.34 ± 1.06	140.42 ± 1.38	143.00 ± 1.41
5	147.61 ± 1.24	143.48 ± 1.64	144.02 ± 1.23
6	145.53 ± 1.24	140.09 ± 1.60	142.84 ± 1.02

Data are presented in EMMs ± standard error.

a *p* < 0.001

A similar pattern was observed for intradialytic BP. Time (Wald *χ*^2^ = 16.468, *p* = 0.006), treatment strategy (Wald *χ*^2^ = 56.512, *p* < 0.001), and its interaction (Wald *χ*^2^ = 29.373, *p* = 0.001) were all significant determinants of intradialytic pressure trajectories. The EMMs revealed a marked decline in intradialytic pressure from month two, with consistently higher values in the combination-therapy group. Compared with combination therapy, significant reductions were present in the SV monotherapy group, beginning at month two (*p* < 0.001), and in the dose-reduction/discontinuation group from months two to four (*p* < 0.05 to *p* < 0.001) (Table 3). Overall, patients who received SV alone or underwent dose reduction/discontinuation exhibited greater improvement in both interdialytic and intradialytic BP, when compared to patients who required additional antihypertensive agents.

**Table 3:** EMMs of intradialytic SBP (mmHg) over six months.

Follow-up months	Combination therapy	SV monotherapy	SV dose reduction/discontinuation
1	153.31 ± 1.18	145.00 ± 5.84	–
2	150.58 ± 1.32	137.06 ± 1.86^c^	131.56 ± 2.04^c^
3	145.88 ± 1.24	140.33 ± 1.80	136.43 ± 1.91^b^
4	143.07 ± 1.45	139.54 ± 2.12	135.48 ± 1.42^a^
5	141.76 ± 1.63	134.12 ± 1.88	135.94 ± 1.32
6	142.27 ± 1.71	132.27 ± 2.25	136.19 ± 1.18

Data are presented in EMMs ± standard error.

a *p* < 0.05

b *p* < 0.01

c *p* < 0.001

In order to assess whether the between-group differences in BP were independent of potential confounders, mixed-effects models were repeated after adjusting for NT-proBNP, interdialytic weight gain/dry weight ratio, diabetes, age, and sex. After adjustment, treatment strategy (interdialytic: Wald *χ*^2^ = 7.038, *p* = 0.030; intradialytic: Wald *χ*^2^ = 9.691, *p* = 0.008) and time (interdialytic: Wald *χ*^2^ = 69.179, *p* < 0.001; intradialytic: Wald *χ*^2^ = 44.038, *p* < 0.001) remained significant determinants of BP, while the treatment strategy×time interaction did not reach statistical significance in either model (interdialytic: *p* = 0.099; intradialytic: *p* = 0.144). These findings suggest that the overall between-group differences in both interdialytic and intradialytic BP were robust to the adjustment for volume status, cardiac function, and key demographic variables (Supplementary Tables 1, 2).

In order to further investigate the outcomes, patients were regrouped at six months into patients who remained on an unchanged SV dose and patients who underwent SV dose reduction or discontinuation. No significant differences were observed between these two groups, in terms of clinical or laboratory parameters, including HF classification, NT-proBNP levels, or echocardiographic measurements (Table 4). These results indicate that the dose reduction or discontinuation of SV at six months did not adversely affect the cardiac function or biomarker profiles.

**Table 4:** Comparison of clinical and laboratory parameters between patients with and without SV dose reduction/discontinuation.

Parameters	Unchanged SV (*n* = 42)	SV reduction/discontinuation (*n* = 109)	*p*
Age	57.95 ± 10.92	59.03 ± 8.803	0.570
Sex			0.941
Male, *n* (%)	24 (57.14)	62 (56.88)	
Female, *n* (%)	18 (42.86)	47 (43.12)	
DM, *n* (%)	24 (57.14)	51 (46.79)	0.280
Alcohol consumption history, *n* (%)	5 (11.90)	16 (14.68)	0.796
Smoking history, *n* (%)	15 (35.71)	27 (24.77)	0.224
PTH	225.7 ± 107.4	209.5 ± 101.8	0.403
Calcium	2.21 ± 0.12	2.18 ± 0.124	0.318
Phosphorus	1.85 ± 0.45	1.99 ± 0.36	0.086
Kt/V	1.34 ± 0.15	1.32 ± 0.125	0.544
Albumin	40.31 ± 2.92	39.26 ± 3.13	0.732
Hemoglobin	114.80 ± 11.02	112.60 ± 13.34	0.677
NT-proBNP (pg/mL)	6,435 ± 2,669	5,797 ± 2,702	0.194
Echocardiographic measurements			
LVEF (%)	59.52 ± 6.552	61.22 ± 4.50	0.131
Aortic root diameter	27.11 ± 2.57	27.27 ± 4.14	0.794
Left atrial diameter	33.64 ± 4.67	35.42 ± 3.74	0.094
Right ventricular diameter	23.02 ± 1.76	23.80 ± 2.24	0.069
IVST	11.64 ± 0.78	12.11 ± 0.94	0.342
LVPWT	11.59 ± 1.23	11.79 ± 0.71	0.480
LVDd	46.83 ± 5.10	45.51 ± 4.74	0.467
E/A	0.97 ± 0.66	0.89 ± 0.53	0.122
EDV	123.50 ± 31.96	121.2 ± 33.63	0.599
Stroke volume	60.54 ± 16.42	62.40 ± 13.56	0.449
Cardiac output	5.17 ± 0.96	5.25 ± 1.76	0.876

SV, sacubitril/valsartan; DM, diabetes mellitus; PTH, parathyroid hormone; Kt/V, the product of dialyzer urea clearance (K) and dialysis session duration (t) divided by the patient's urea distribution volume (V); LVEF, left ventricular ejection fraction; IVST, interventricular septal thickness; LVPWT, left ventricular posterior wall thickness; LVDd, left ventricular end-diastolic diameter; E/A, ratio of early (E) to late (A) diastolic filling velocities; EDV, end-diastolic volume.

### Hypotension events and clinical management

Among the 151 patients, 70 (46.36%) patients experienced IDH. Across the cohort, a total of 45,597 dialysis sessions were performed (302.0 ± 14.1 sessions per patient). IDH occurred at an average frequency of 3.44 events per patient, corresponding to an overall incidence of 1.14% of all dialysis sessions. Among the 70 affected patients, a cumulative of 23,983 dialysis treatments were delivered, and IDH was detected at an average of 7.43 episodes per patient, yielding an incidence of 2.17% of sessions within this subgroup.

Interdialytic hypotension occurred in one patient, who was managed by discontinuing antihypertensive agents, restricting sodium intake, controlling interdialytic weight gain, and converting vascular access from native arteriovenous fistula to tunneled cuffed central venous catheter due to recurrent thrombosis and access failure.

Post-dialysis orthostatic hypotension, which occurred within 30 min after treatment, was documented in 14 patients. Acute treatment consisted of saline infusion or hypertonic glucose bolus, while long-term preventive approaches were consistent with those applied for IDH.

### Independent risk factors of IDH

In order to identify the independent risk factors for IDH, univariate and multivariable logistic regression analyses were performed. DM (OR: 3.085, 95% CI: 1.137–8.376, *p* = 0.027), higher pre-dialysis NT-proBNP levels (OR: 1.870, 95% CI: 1.109–3.152, *p* = 0.019; continuous data categorized), and greater interdialytic weight gain relative to dry weight (OR: 4.083, 95% CI: 1.763–9.457, *p* = 0.001; continuous data categorized) were independently associated with IDH, while SV use was not a risk factor for IDH (Table 5).

**Table 5:** Univariate and multivariate logistic regression analyses of risk factors for IDH.

Variables	Univariate analysis		Multivariate analysis	
	t/*X^2^*	*P*	OR (95% CI)	*p*
Age	0.094	0.756	-	-
Sex	2.620	0.106	-	-
DM	33.685	<0.001	3.085 (1.137−8.376)	0.027
Pre-dialysis NT-proBNP (categorized)	39.342	<0.001	1.870 (1.109−3.152)	0.019
Pre-dialysis NYHA class				
I	-	-	-	-
Ⅱ	0.163	0.374	-	-
Ⅲ	1.297	0.255	-	-
Ⅳ	11.469	<0.001	-	-
SV use	1.268	0.997	-	-
Alcohol consumption history			-	-
Smoking history	2.689	1.101	-	-
Ratio of interdialytic weight gain to dry weight (categorized)	36.766	<0.001	4.083 (1.763−9.457)	0.001
PTH	2.036	0.048	-	-
Calcium	1.013	0.248	-	-
Phosphorus	0.749	0.458	-	-
Kt/V	0.084	0.542	-	-
Albumin	3.315	0.045	-	-
Anemia	1.832	0.316	-	-

The ratio of interdialytic weight gain to dry weight=(interdialytic weight gain/dry weight) × 100%. Continuous variables (NT-proBNP and weight gain ratio) were categorized according to clinically relevant thresholds for regression analysis. OR, odds ratio; CI, confidence interval; DM, diabetes mellitus; NT-proBNP, N-terminal pro B-type natriuretic peptide; NYHA, New York Heart Association; SV, sacubitril/valsartan; PTH, parathyroid hormone; Kt/V, the product of dialyzer urea clearance (K) and dialysis session duration (t) divided by the patient's urea distribution volume (V).

### Changes in HF-related indicators during follow-up

HF-related indices, including the NYHA functional class, NT-proBNP, and echocardiographic parameters, were compared between baseline and at the six-month observation period. Prior to dialysis initiation, 43 (28.48%) patients were classified as NYHA class II, 68 (45.03%) patients were classified as class III, and 21 (13.91%) patients were classified as class IV. The NYHA functional class significantly improved after treatment. The NT-proBNP levels markedly decreased from 11,680 (7,640–20,000) pg/mL at baseline to 4,830 (4,310–6,980) pg/mL at follow-up (*p* < 0.001). The echocardiography revealed significant increases in LVEF and cardiac output, along with reductions in aortic root diameter, right ventricular diameter, and left ventricular end-diastolic dimension (all, *p* < 0.001). The other cardiac structural parameters, including interventricular septal thickness, posterior wall thickness, E/A ratio, end-diastolic volume, and stroke volume, remained unchanged (Table 6).

**Table 6:** Comparison of HF-related parameters before initiating HD and at the six-month observation period.

Parameters	Before HD	Follow-up	t/Z	*p*
NT-proBNP (pg/mL)	11,680 (7,640, 20,000)	4,830 (4,310, 6,980)	10.589	<0.001
NYHA functional class				
I	19 (12.58%)	66 (43.71%)		
II	43 (28.48%)	69 (45.70%)	8.820	<0.001
III	68 (45.03%)	16 (10.59%)		
IV	21 (13.91%)	0		
Echocardiographic measurements				
LVEF	60.2 (55, 62.6)	62.3 (58.6, 64.3)	8.104	<0.001
Aortic root diameter	28.3 (26.8, 31.1)	26.8 (25.9, 28.9)	5.876	<0.001
Left atrial diameter	36.8 (34.9, 38.8)	34.5 (32.7, 37.5)	6.763	<0.001
Right ventricular diameter	24.15 ± 2.07	22.91 ± 1.68	6.943	<0.001
IVST	12.02 ± 0.95	11.99 ± 0.91	0.279	0.781
LVPWT	11.8 (11.3, 12.5)	11.6 (11.2, 12.5)	0.694	0.488
LVDd	45.8 (43.9, 49.6)	45.1 (43.1, 48.9)	2.127	0.033
E/A	0.9 (0.7, 1.1)	0.9 (0.7, 1.2)	0.529	0.597
EDV	120 (106, 135)	114 (106, 135)	1.608	0.108
Stroke volume	64 (54.1, 69)	64.4 (54.6, 69)	0.212	0.832
Cardiac output	4.7 (3.8, 5.1)	5.1 (4.4, 5.9)	7.651	<0.001

HD, hemodialysis; NT-proBNP, N-terminal pro B-type natriuretic peptide; NYHA, New York Heart Association; LVEF, left ventricular ejection fraction; IVST, interventricular septal thickness; LVPWT, left ventricular posterior wall thickness; LVDd, left ventricular end-diastolic diameter; E/A, ratio of early (E) to late (A) diastolic filling velocities; EDV, end-diastolic volume.

### Incidence of HF events

During the six-month observation period, 19 patients experienced HF episodes, yielding an estimated cumulative incidence of 12.58% at the last follow-up. All episodes were characterized by peripheral edema, pulmonary congestion, and elevated NT-proBNP levels, and were preceded by rising BP. Among patients treated with SV either alone or in combination, six patients developed composite major adverse outcomes (four HF hospitalizations and two cardiovascular deaths). In the dose-reduced or discontinued SV group, 13 composite events occurred (11 HF admissions and two cardiovascular deaths). No statistically significant difference was observed between groups (*p* > 0.05) (Table 7).

**Table 7:** Comparison of cardiovascular hospitalization and mortality between patients maintained on full-dose SV and patients with dose reduction or discontinuation.

Outcome	Unchanged SV (*n* = 42)	SV reduction/discontinuation (*n* = 109)	*p*
HF hospitalizations	4	11	0.741
Cardiovascular deaths	2	2	0.327
Total	6	13	0.695

## Discussion

The present retrospective study demonstrated that the dose reduction or discontinuation of SV is common in patients undergoing maintenance HD, particularly in the first few months after dialysis initiation. Patients who continued full-dose SV with other antihypertensive agents had higher interdialytic and intradialytic BP, when compared with patients on SV monotherapy or reduced/discontinued therapy. Notably, SV dose reduction or discontinuation was not associated with worsening HF status, NT-proBNP levels, or echocardiographic parameters at six months. Furthermore, DM, elevated NT-proBNP, and greater interdialytic weight gain relative to dry weight were identified as independent predictors of IDH, while SV use itself was not identified as an independent predictor of IDH.

Previous studies have consistently demonstrated the potent antihypertensive effects of SV. A meta-analysis that included 1,597 patients reported that SV therapy reduced SBP by an average of 11.09 mmHg and DBP by 4.37 mmHg, with significantly greater BP reduction, when compared with conventional therapy (19). Wen et al. also observed the dose-dependent BP-lowering effect of SV, with patients receiving the target dose (200 mg twice daily), and achieving greater reductions, when compared to patients receiving lower doses (20). In contrast, the tolerable dose of SV in patients undergoing HD was generally lower, when compared to that in non-dialysis populations, with maintenance doses typically ranging from 100 to 150 mg twice daily. This limitation may be attributed to the increased risk of IDH. In the present study, the patient-level incidence of IDH was 46.36%. The seemingly low incidence reflects the calculation on a per-dialysis-session basis, whereby only a small proportion of total dialysis sessions were complicated by IDH events. This distinction between patient-level and session-level incidence may account for the discrepancy. Consistent with these observations, the present study revealed that the SV doses gradually decreased over time in HD patients, and the maintained doses were lower than those reported in the aforementioned studies. This trend may be explained by local BP management strategies that emphasized volume control through intensified ultrafiltration and sodium removal, resulting in further BP reduction and the subsequent patient-initiated dose reduction of SV (21). Importantly, SV use itself was not identified as an independent risk factor for IDH in the present multivariate analysis. Instead, IDH was more strongly associated with DM, higher pre-dialysis NT-proBNP levels, and greater interdialytic weight gain relative to dry weight. These findings suggest that patients with impaired cardiac reserve and excessive volume burden are more susceptible to IDH, rather than the direct pharmacologic effects of SV. However, important dialysis-related parameters, including ultrafiltration rate, dialysate composition, and dialysate temperature, were not included in the model. Therefore, a potential association between SV use and IDH cannot be completely excluded.

SV treatment improves cardiac structure and function in HD patients, including reductions in NT-proBNP levels, decreases in LVEDd, increases in LVEF, and improvements in diastolic function indices, such as the E/e′ ratio (11, 22). In a single-center prospective study, Chen et al. observed that SV therapy was associated with improvements in LVEF and arteriovenous fistula blood flow among HD patients with HFrEF (23). These findings are consistent with the pharmacologic mechanisms of SV, which enhances the natriuretic peptide system to reduce preload, while simultaneously suppressing the renin-angiotensin-aldosterone system activation to decrease afterload, thereby improving overall cardiac performance (24). However, most of these studies were limited by small sample sizes. In the present study, patients were stratified according to SV dose adjustment, and no significant differences in cardiac structural or functional parameters were observed between patients who continued SV therapy and patients who reduced or discontinued treatment.

SV has demonstrated potential benefits in reducing cardiovascular morbidity among HD patients. A large observational study (25) based on the United States Renal Data System, which included 1,434 SV users and 29,654 angiotensin-converting enzyme inhibitor or angiotensin receptor blocker users, revealed that SV use was associated with an 18% reduction in all-cause mortality and a 14% reduction in all-cause hospitalization. However, no significant difference in cardiovascular mortality was observed. In contrast, several smaller single-center studies, including the retrospective study conducted by Ding et al. (26) and the prospective study conducted by Huang et al. (27), did not observe significant improvements in mortality or HF-related hospitalization. These discrepancies may be attributable to the limited sample sizes and relatively short follow-up durations. In the present study, no significant differences in HF hospitalization or cardiovascular mortality were observed between patients who continued SV therapy and patients who underwent dose reduction or discontinuation, despite the higher number of HF-related events in the dose-reduction/discontinuation group (13 vs*.* 6). This imbalance may be attributable to limited sample size and relatively short follow-up duration, which reduced the statistical power to detect between-group differences. In addition, patients in the dose-reduction/discontinuation group may have had a more complex clinical profile, including lower BP, greater volume fluctuations, and higher burden of comorbidities, all of which could contribute to a higher observed event rate as potential confounders. Therefore, these findings should not be interpreted as evidence that SV dose reduction or discontinuation leads to worse outcomes. In addition, the lack of significant changes in echocardiographic parameters, such as the E/A ratio, end-diastolic volume, and stroke volume, may be explained by the limited sample size and measurement variability, rather than the true absence of physiological effect. Overall, these results suggest that SV dose adjustment in HD patients should be undertaken with careful clinical monitoring, and larger prospective studies are required to clarify the relationship between SV dosing and HF outcomes in this population.

In real-world clinical practice, as dialysis duration increases, nonpharmacologic factors, such as ultrafiltration strategies, volume status control, and dry weight adjustment, play an increasingly dominant role in BP regulation and cardiac function among patients undergoing HD (28). In the present cohort, the progressive improvements in cardiac function, declining BP, and delayed adjustment of dry weight frequently led to the reduction or discontinuation of antihypertensive medications with potent BP-lowering effects, and SV was often the first agent withdrawn. It remains uncertain whether the discontinuation of SV would result in loss of its cardioprotective effects or adversely affect long-term cardiovascular outcomes. Within the relatively limited follow-up period of the present study, SV dose reduction or discontinuation was not associated with the worsening HF, deterioration in echocardiographic parameters, or increases in NT-proBNP levels. These findings suggest that in the short term, meticulous dialysis management, including accurate dry weight assessment, appropriate ultrafiltration volumes, and strict sodium and fluid restriction, may exert a greater influence on BP control and cardiac function, when compared to pharmacologic therapy alone. Clinically, the present results indicate that not all patients undergoing maintenance HD with hypertension necessarily require long-term treatment with target doses of SV.

The present study had several limitations. First, subgroup analyses according to HF phenotype were not performed, which limited the ability of the study to determine whether specific patient subgroups derive greater benefits from SV therapy. Second, the retrospective design and single-center setting may have limited the generalizability. For instance, inclusion was restricted to patients who initiated SV therapy before dialysis initiation, and continued treatment after starting HD. This design reflected a common real-world practice pattern in the center of the investigators, where many patients with advanced CKD were already receiving SV for heart failure or refractory hypertension prior to dialysis initiation. However, this criterion may have selected a relatively specific subgroup of HD patients, and may have limited the applicability of the present findings to patients who initiated SV only after starting maintenance dialysis. Third, the primary etiology of ESRD was not systematically analyzed, because many patients presented at advanced CKD stage 5 with incomplete historical records and without kidney biopsy confirmation, limiting the accurate classification of underlying renal disease. Fourth, no formal sample size or power calculation was performed, particularly for the subgroup analyses. Therefore, the possibility of a type II error cannot be excluded. Future large-scale, multicenter, prospective studies are needed to clarify the optimal role, dosing strategies, and long-term safety of SV in patients undergoing HD, while minimizing the risk of dialysis-related hypotension and other adverse events.

## Conclusion

SV treatment was associated with BP reduction in HD patients, as well as improvements in left ventricular function and cardiac remodeling. It may also be associated with a reduced risk of cardiovascular hospitalization. Given the retrospective observational nature of this study, these findings should be interpreted with caution, and clinicians should carefully balance the potential risks and benefits while closely monitoring patients receiving this therapy. Larger, randomized controlled trials are needed to assess the long-term efficacy and safety of SV in HD patients with different HF phenotypes and to establish optimal dosing and monitoring protocols.

## Data Availability

The original contributions presented in the study are included in the article/Supplementary Material, further inquiries can be directed to the corresponding author.
