# Supplementary material for: Dose reduction and discontinuation of sacubitril/valsartan over time in patients undergoing maintenance hemodialysis
Source: Front Cardiovasc Med. 2026 Aug 12;13:1804910. doi: 10.3389/fcvm.2026.1804910 (PMC13506787; doi:10.3389/fcvm.2026.1804910)
Supplement: Supplementary file 1 [file Table1.docx]

**Supplementary Table 1. Adjusted EMMs of interdialytic SBP (mmHg) over six months**

| **Follow-up months** | **Combination therapy** | **SV monotherapy** | **SV dose reduction/discontinuation** |
| --- | --- | --- | --- |
| 1 | 150.90 ± 1.07 | 147.27 ± 1.90 | -- |
| 2 | 147.06 ± 1.10 | 144.91 ± 1.84 | 145.07±2.59 |
| 3 | 144.27 ± 1.18 | 141.23 ± 1.49 | 143.86±1.56 |
| 4 | 141.68 ± 1.07 | 141.59 ± 1.82 | 145.53±1.37 |
| 5 | 144.22 ± 1.35 | 142.93 ± 1.72 | 145.64±1.12 |
| 6 | 142.29 ± 1.29 | 135.75 ± 2.14 | 144.15±1.00^a^ |

**Notes:** Models adjusted for NT-proBNP, interdialytic weight gain/dry weight ratio, diabetes, age, and sex. Data are presented in EMMs ± standard error. **^a^***p*<0.05

**Supplementary Table 2. Adjusted EMMs of intradialytic SBP (mmHg) over six months**

| **Follow-up months** | **Combination therapy** | **SV monotherapy** | **SV dose reduction/discontinuation** |
| --- | --- | --- | --- |
| 1 | 153.21 ± 1.18 | 148.81 ± 6.35 | -- |
| 2 | 149.06 ± 1.30 | 143.03 ± 2.72 | 141.44 ± 2.31 |
| 3 | 144.08 ± 1.18 | 142.91 ± 1.92 | 139.49 ± 1.75 |
| 4 | 139.83 ± 1.41 | 140.44 ± 2.39 | 137.77 ± 1.33 |
| 5 | 139.34 ± 1.55 | 134.34 ± 1.69 | 136.93 ± 1.20 |
| 6 | 139.89 ± 1.68 | 129.91 ± 2.35 | 137.08 ± 1.13 |

**Notes:** Models adjusted for NT-proBNP, interdialytic weight gain/dry weight ratio, diabetes, age, and sex. Data are presented in EMMs ± standard error.
